# Population Size Estimation of Men Who Have Sex with Men through the Network Scale-Up Method in Japan

**DOI:** 10.1371/journal.pone.0031184

**Published:** 2012-01-27

**Authors:** Satoshi Ezoe, Takeo Morooka, Tatsuya Noda, Miriam Lewis Sabin, Soichi Koike

**Affiliations:** 1 Evidence, Strategy and Results Department, The Joint United Nations Programme on HIV/AIDS (UNAIDS), Geneva, Switzerland; 2 Department of Public Health, Juntendo University Graduate School of Medicine, Tokyo, Japan; 3 Department of Health Service Management, Graduate School at International University of Health and Welfare, Tokyo, Japan; 4 Department of Community Health and Preventive Medicine, Hamamatsu University School of Medicine, Hamamatsu, Japan; 5 Department of Planning, Information and Management, The University of Tokyo Hospital, Tokyo, Japan; Children's Hospital of Eastern Ontario, Canada

## Abstract

**Background:**

Men who have sex with men (MSM) are one of the groups most at risk for HIV infection in Japan. However, size estimates of MSM populations have not been conducted with sufficient frequency and rigor because of the difficulty, high cost and stigma associated with reaching such populations. This study examined an innovative and simple method for estimating the size of the MSM population in Japan. We combined an internet survey with the network scale-up method, a social network method for estimating the size of hard-to-reach populations, for the first time in Japan.

**Methods and Findings:**

An internet survey was conducted among 1,500 internet users who registered with a nationwide internet-research agency. The survey participants were asked how many members of particular groups with known population sizes (firepersons, police officers, and military personnel) they knew as acquaintances. The participants were also asked to identify the number of their acquaintances whom they understood to be MSM. Using these survey results with the network scale-up method, the personal network size and MSM population size were estimated. The personal network size was estimated to be 363.5 regardless of the sex of the acquaintances and 174.0 for only male acquaintances. The estimated MSM prevalence among the total male population in Japan was 0.0402% without adjustment, and 2.87% after adjusting for the transmission error of MSM.

**Conclusions:**

The estimated personal network size and MSM prevalence seen in this study were comparable to those from previous survey results based on the direct-estimation method. Estimating population sizes through combining an internet survey with the network scale-up method appeared to be an effective method from the perspectives of rapidity, simplicity, and low cost as compared with more-conventional methods.

## Introduction

HIV/AIDS is a major global health challenge, with 33.3 million people estimated to be living with HIV as of the end of 2009, according to the Joint United Nations Programme on HIV/AIDS (UNAIDS) [1]. In Japan, the estimated number of people living with HIV is 8,100 in a country with a total population of 127.5 million as of 2009 [2]. Although this figure translates to a low-level epidemic, with an estimated HIV prevalence of less than 0.01%, the number has increased from 6,500 in 2001. Moreover, the number of people newly infected with HIV in 2008 was 1,126 and has constantly been increasing for the last two decades. The majority of HIV cases among those newly infected in 2008 were as follows: 88.7% were as a result of sexual contact, of which 78.0% were associated with behaviors among men who have sex with men (MSM); less than 2% were associated with injection drug use and mother-to-child transmission [3].

When designing effective policy interventions for public health issues, it is crucial to identify the size of the high-risk populations and the epidemiological trends within those populations. In particular, HIV responses at a national level can be strengthened by better information on the number of people in a given population who engage in behaviors that increase their risk for HIV. By estimating the size of the populations at higher risk for HIV, a country can revise its strategic plans, allocate resources appropriately, improve the modeling of its epidemic, and advocate for services for those populations [4]. Unfortunately, the populations most at risk for HIV infection, such as sex workers, people who inject drugs, and MSM, are often hard to reach and thus present significant size-estimation challenges. The global data on MSM population sizes, for example, is insufficient, especially in low- and middle-income countries [2], [5]. For these reasons, UNAIDS, WHO and their partners have explored various methods for estimating the size of key hard-to-reach populations, including MSM, that are at high risk for HIV [6].

In the case of Japan, estimating the population size of MSM is crucial in the AIDS response considering the important contribution that HIV risk behaviors associated with MSM have on HIV transmission in the country. Two major surveys have previously been conducted in Japan. A self-completed, mail-in survey in 1991 found an MSM prevalence (respondents who reported having had sex with men out of all male respondents) of 1.2% among the male population [7] while a field survey in 1999 found that of 1.5% [8]. However, data on the population size of MSM has not been properly updated in a rigorous manner.

Although determining the true prevalence of MSM behavior and thus the potential true size of the MSM population is desirable, the high cost and difficulty of approaching hidden populations have prevented policy makers and the research community from thoroughly and frequently conducting such methods in Japan. In light of the importance of obtaining a reliable and up-to-date estimate of the size of the MSM population, which has the highest risk of acquiring HIV infection in Japan, this study examined the use of an innovative and simple method for estimating the size of the MSM population. It is the first Japanese study to combine the network scale-up method, which is increasingly used in public health to estimate the size of hard-to-reach populations [9], with an internet survey.

## Methods

### The network scale-up method

The network scale-up method is a social network method for estimating the size of hard-to-reach populations. There are 3 steps in the network scale-up method. The method first assumes that [m/c = e/t], where (m) equals the number of individuals in a certain subpopulation that a person knows, (c) equals the total number of people that the person knows (the personal network size), (e) equals the size of a subpopulation of known size, and (t) equals the size of the total population. The second step is to compute (c) based on the equation [c = m*t/e]. The known size of the subpopulation (e.g., the total number of firepersons in Japan) is used as (e), the number of members of that specific subpopulation (e.g., firepersons) that a given individual knows is used as (m), and the total population (e.g., the population of Japan) is used as (t). The third step is to compute a national prevalence estimate of the group of interest (e.g., MSM) by dividing the number of members of the group with an unknown prevalence that an individual knows by (c). This method is applied to the epidemiological surveys of hard-to-reach populations when more direct methods are difficult to apply.

### Internet survey

The Internet was estimated to be accessible to 78.0% of the population and had 94 million users in 2009 [10]. Owing to this large proportion, Owing to this large proportion, the Internet enables researchers to engage numerous participants in a short period of time at low cost, allowing access to populations that cannot be reached using conventional sampling procedures. As a result, Internet surveys have been increasingly applied when conducting behavioral surveys of hard-to-reach or hidden populations in Japan [11]. Thus, in this study, we tested the network scale-up method through an Internet survey.

The survey was conducted among the first 1,500 respondents, who were made proportionate to the national population distribution by sex and age groups according to the 2005 Japanese census. They were invited to participate in the survey from a large survey panel managed by a major nationwide internet research agency called *Intage*. The overall size of the survey panel at the time of survey was 721,877 people, of whom 386,133 were male (53.5%). The age distribution of the survey panel was 10–19 (1.0%), 20–29 (16.9%), 30–39 (39.2%), 40–49 (27.7%), 50–59 (11.1%), and 60–69 (4.2%). The survey panel consisted of people initially recruited through a web site managed by the research agency. At the time of registration, they were required to provide information such as sex, age, profession, place of living, and to agree that they would be asked to participate in different market research surveys. The research agency could approach the panel for certain surveys based on these data. The identification data including sex and age of the survey panel participants were verified directly by the research agency every time they participate in a survey.

In this survey, the respondents were asked how many members of particular subgroups with known population sizes they knew. The respondents were also asked how many MSM they knew. Target numbers of respondents were set for each sex and age category to ensure that these distributions would be proportionate to the national population distribution from the 2005 Japanese census. The survey requests were sent by the research agency to the panelists who were randomly selected by each sex and age category. The panelists who consented to participate in the survey accessed the designated website upon verification of their personal information and responded to the survey. The participants had the option to not respond to any part of the questionnaire and the option to discontinue the survey at any point. The survey was closed as the target numbers of respondents for each sex and age category were met. The first 1,500 valid responses of the 6,332 panelists who were asked to participate in the survey were obtained and analyzed. The survey was conducted from February 13^th^ to 17^th^, 2009.

### Ethics Statement

The survey was conducted by a research agency contracted by the study group. At the stage of releasing the data to researchers, personal identifiers (names, full addresses) were stripped from the dataset. The study was reviewed and approved by the Research Ethics Committee, Graduate School of Medicine and Faculty of Medicine, The University of Tokyo.

### Estimation of personal network size using subgroups with known size

The specific subgroups with known sizes that have commonly been used in previous studies conducted in the US include individuals with a specific first name, people born in a given time period, those with distinct occupations such as postal workers, pilots, and, most commonly, individuals with specific last names. Subgroups with specific first or last names were not used in this study, however, because the prevalence of last names differs among localities, and the prevalence of first names differs by age groups in Japan; therefore, proper adjustment for this variability in estimating personal network size was not considered to be feasible.

To identify the appropriate subgroups with known sizes to be used in this study, a pilot survey was conducted during the period from 6 to 10 February 2009. The first 225 respondents among the same survey panel were asked about how many members of 10 subgroups they knew. These subgroups, the average number known, known population size and population size based on backward estimation (that is, to treat population size as unknown and estimate it using the average network size (c), total population (t) and the average number known (m)) by each subgroup are listed in Table 1.

**Table 1:** The result of the pilot study to determine subgroup to be used in the study (N = 225).

Subgroup with known size	Average number known	Known population size**	Population size based on backward estimation
Fireperson*	0.34	156013	123925
Police officer*	0.67	252764	239244
Postal worker	0.87	111253	311534
Military personnel*	0.39	230291	139416
Physician	2.17	286699	779695
Person with baby born within last one year	1.30	1062530	466440
Twins	0.56	1468488	203099
Person deceased by cancer	0.60	344105	215148
Person deceased by stroke	0.11	127041	39587
Person with motorcycle license	3.90	2265858	1404483

*Used in this study.

**Data on known population size are from Fire and Disaster Management Agency; National Police Agency; Ministry of Internal Affairs and Communication; Ministry of Defense; and Ministry of Health, Labour and Welfare.

Based on the pilot study results, we excluded those subgroups which met either of the following criteria: 1) if a major discrepancy existed between the known population size and the population size derived from backward estimation, 2) if transmission error was likely and the adjustment was not considered to be feasible.

After examining the first condition, postal worker, physician, person with baby born within last one year, twins, person deceased by stroke were excluded because the known population size and the population size derived from backward estimation differed considerably. And after examining the second condition, person deceased by cancer was excluded because the Japanese tend not to reveal cancer status even to acquaintances. Also, person with motorcycle license was excluded because the information of possession of motorcycle license is rather trivial therefore not often discussed even among acquaintances. These transmission errors were considered to have significant effects on the known population but the adjustments were not thought to be feasible. As a result, firepersons, police officers, and military personnel were selected to be used in the actual study.

To estimate their average network size, the respondents were asked to specify the numbers of acquaintances (including the number of male acquaintances) who were active-service members of the following subpopulations: firepersons (total: 156,013, male: 153,626) [12], police officers (total: 252,764, male: 239,240) [13], and military personnel (described as “Self Defense Force personnel” in the survey) (total: 230,291, male: 219,051) [14]. Similarly, the respondents were asked to identify the number of their acquaintances whom they understood to be MSM.

The working definition of “acquaintance” was formulated based on the existing definition by Killworth et al.: “mutually recognize each other by sight or name, can be contacted, and have had contact within the last two years, either in person, by phone or mail” [15]. An acquaintance was thus a person who met all of the following criteria: 1) a person other than oneself, including family and relatives; 2) a person one has either met directly or can recognize by name (including first, last or “handle” name); 3) a person who can be contacted directly; 4) a person with whom one has had contact within the last two years, either in person, by phone or mail (including internet communication such as e-mail); and 5) a person who lives in Japan.

### Estimation of MSM population size adjusted for the transmission error

To minimize “transmission error”, which includes the potential underestimation from not all of the respondents who fit the definition of MSM being likely to have fully come out, an adjustment was made using the best available data on the average number of acquaintances to whom MSM have come out divided by the average total number of persons in their personal network (we call this “the coming-out rate” in this article) in Japan. According to a previous survey that examined HIV prevention behavior among MSM [16], the average number of acquaintances to whom MSM have come out was determined to be 5.09, of whom an average of 0.21 were parents and 4.88 were other acquaintances. This previous survey was conducted from August 11^th^ to November 30^th^, 2005. It had 5,731 valid responses (obtained through the internet) from men who had ever had sex with men. All of the survey respondents were anonymous and electronically confirmed that they agreed to the purpose and methods of the survey before they responded to the questionnaires.

Thus, “the coming-out rate” for MSM among people they know was computed by dividing the average number of acquaintances to whom the MSM had come out (5.09) by the previously mentioned network size (c). The prevalence of MSM among the total male population in Japan was then estimated by dividing the rate of MSM in the average personal male network by this MSM “coming-out rate”.

## Results

### Characteristics of the participants

There were 1,500 participants in the study, of whom 725 were male and 775 were female. The average age ± standard deviation was 50.8±16.9 for all the participants, 50.4±17.5 for the males and 51.2±16.3 for the females. The percentages by sex and age groups were made proportionate to those of the Japanese population (Table 2).

**Table 2:** The characteristics of study participants.

	Male		Female		Total	
Age Group	number	%	Number	%	number	%
20–29	104	6.9	99	6.6	203	13.5
30–39	132	8.8	129	8.6	261	17.4
40–49	114	7.6	113	7.5	227	15.1
50–59	124	8.3	125	8.3	249	16.6
≥60	251	16.7	309	20.6	560	37.3
Total	725	48.3	775	51.7	1,500	100.0
Average ± S.D.*	50.4±17.5		51.2±16.3		50.8±16.9	

*S.D.: Standard Deviation.

### Estimation of personal network size

**Figure 1:**
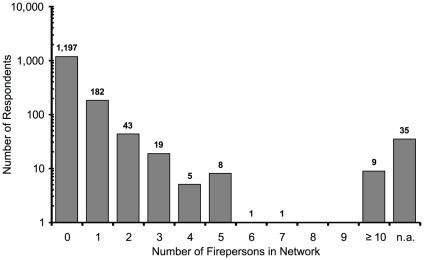
Firepersons.

**Figure 2:**
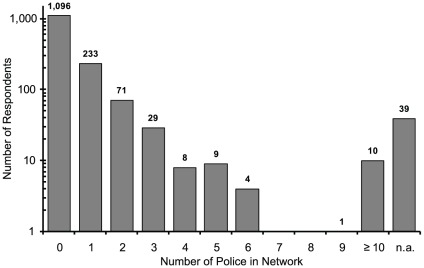
Police Officers.

**Figure 3:**
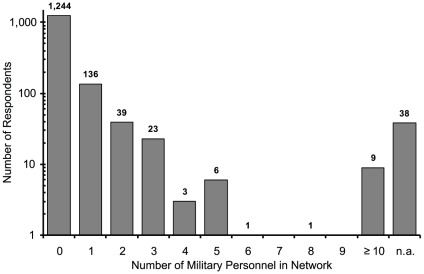
Military Personnel.

Figures 1, 2, 3 indicate the distributions of the number of acquaintances who were firepersons, police officers, and military personnel. The total numbers of valid responses for firepersons, police officers, and military personnel were 1,464, 1,460, and 1,461, respectively. The personal network size (c) was then estimated by computing [c = m*t/e], using (t) = 127.5 million as the total Japanese population, (e) = the actual total populations of firepersons, police officers, and military personnel, and (m) = the average number of acquaintances who were firepersons, police officers, and military personnel per respondent. The personal network sizes based on the average number of acquaintances who were firepersons, police officers, and military personnel were 417.6, 337.5, and 335.4, respectively, regardless of sex, and 201.4, 162.1, and 158.4, respectively, for males only. Thus, the personal network sizes using the averages of these two sets of three numbers were 363.5 regardless of sex, and 174.0 for male only. The former was used to calculate “the coming-out rate” and the latter was used to calculate the MSM prevalence among male.

### Estimation of MSM population size

**Figure 4:**
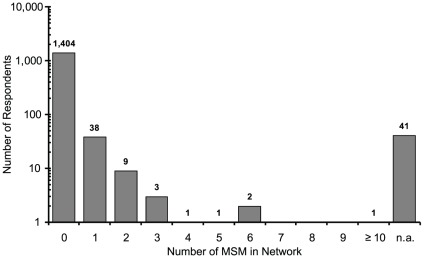
MSM.

Figure 4 indicates the distribution of the number of acquaintances who were MSM. The proportion of MSM in personal male networks was 0.0402%. Because the average number of acquaintances to whom the MSM had come out was 5.09 and the average personal network size was 363.5, “the coming-out rate” for the MSM among the people they knew was computed to be 5.09/363.5 = 1.40%. Thus, by dividing the proportion of MSM in a personal male network (0.0402%) by “the coming-out rate” of 1.40%, the prevalence of MSM in the total male population was estimated to be 2.87%.

### Backward estimation

To check on the reliability of the above results, as used in the pilot study to select the subgroups with known sizes, we conducted backward estimation, where population size was treated as unknown and estimated it using the average network size (c), total population (t) and the average number known (m). As in Table 3, discrepancies between known population size and population size based on backward estimation were not considerable; therefore, the study results were considered to be reliable.

**Table 3:** Comparison between known population size and population size based on backward estimation (N = 1500).

Subgroup	Sex	Average number known	Known population size*	Population size based on backward estimation
Fireperson	All	0.51	156013	179216
	Male only	0.50	153626	177834
Police officer	All	0.67	252764	234725
	Male only	0.62	239240	222901
Military personnel	All	0.61	230291	212476
	Male only	0.56	219051	199494

*Data on known population size are from Fire and Disaster Management Agency; National Police Agency; and Ministry of Defense.

## Discussion

### Estimation of hard-to-reach populations by Internet-based scale-up method

The estimated personal network size of 363.5 in this study was not very far from estimates from prior studies in Japan, which found network sizes of 206.21 (standard error: 9.85) in urban areas and 196.55 (standard error: 10.91) in rural areas [17]. The estimated MSM prevalence of 2.87% among the total male population based on the estimated network size in Japan that was obtained by this study was also comparable to results published previously. This outcome suggests the validity of Internet-based scale-up methods for estimating the MSM population size. This method may be applicable to other settings in need of MSM population size estimation and to estimating hard-to-reach populations other than MSM.

### Limitations of this study

The network scale-up method assumes three conditions; therefore, the accuracy of the estimate depends on the validity of these assumptions. The first assumption is that all members of the population (t) (e.g., the total population in Japan) have an equal chance of being acquainted with members of the group of interest with unknown size (e) (e.g., MSM in Japan). The second assumption is that everyone has perfect information about their acquaintances. The third assumption is that the respondents can accurately recall the number of acquaintances they have and provide this information in a short period of time. The biases arising from deviations from these three assumptions are called barrier effects, transmission error, and estimation effects, respectively [18].

Additionally, Internet surveys are subject to several biases. First, there can be a reporting bias (sensitive data, such as sexual orientation, are often underreported). Second, there are age biases among the users, where younger users tend to have more access [19]. Thirdly, there are selection biases (convenience sample) because the samples are made up of volunteer Internet users [20]. Finally, there could be a bias among respondents who may have more available time to participate in an Internet survey if it is a first-come-first-serve enrollment.

However, the benefits of low cost and rapid response continue to make the use of Internet surveys a valuable method, particularly in settings, such as Japan, where the Internet is widely used by the majority of the population. The risk of overlapping or fictitious respondents can be avoided by verifying the identities of the respondents when they answer the survey. Additionally, as in this study, the number of respondents can be adjusted so that their distribution by sex and age categories is proportionate to that of the total population.

The questionnaires used in the network scale-up method could have a certain degree of ambiguity. For example, the definitions of “MSM,” “homosexual,” “acquaintance”, and “personal network” are not always consistent; however, every attempt was made in this survey to minimize confusion by defining these terms for the survey respondents. Additionally, the numbers of members of groups with large populations tend to be under-reported, and the numbers of members of groups with smaller populations tend to be over-reported [15], [21].

Preferences for reporting certain numbers may also affect the accuracy of the estimation. McCarty et al. [18] have observed the tendency of respondents to report numbers in which the last digit is 0 or 5, especially when the number is more than 10, although this bias has minimal impact on the accuracy of estimation. Asking about sensitive subpopulations could lead to the underreporting of the members of such subpopulations as well, resulting in the underestimation of the corresponding network sizes. In a prior study to estimate the number of heroin users at 14 US sites, for example, Kadushin has argued that the reason the estimated network size was fairly small (55) was underreporting due to sensitivity about this particular subpopulation [22].

In addition, the affiliation of the respondent with a specific subpopulation has a direct impact on the estimation of the network size. For example, when a respondent has an affiliation with a certain subpopulation, the respondent has a much greater chance of getting to know members of that subpopulation (and vice versa). Additionally, factors such as the selection of subgroups with known sizes and respondents with large network sizes, such as religious leaders, politicians, corporate managers, and diplomats, may affect personal network size. Also, the number of subgroups with known sizes would affect the estimated network size. Although the three subgroups with known sizes used in this study were carefully selected, more subgroups should be reasonably identified and used to increase the reliability of the network size estimation.

The estimated MSM prevalence of 2.87% was comparable to previous estimates for Japan. However, the accuracy of the estimate is not adequately quantified by providing a confidence interval because we could not reasonably assume “the true distribution of the number of MSM among one's acquaintances” or “the true distribution of the proportion of MSM who come out among their acquaintances”. Also, the MSM “coming-out rate” could only be a point estimate as it was derived from secondary data without a confidence interval.

The accuracy of the assumption that MSM have similar network sizes as other subpopulations could be a source of uncertainty regarding the estimate. In fact, Zheng et al. [23] found that Americans vary greatly in their number of acquaintances; therefore, the network size of MSM could differ greatly from other subgroups used in this study. Also, it was suggested in the same study that Americans show great variation in propensity to form ties to people in some groups, but little variation for other groups.

Lastly, other notable statistical developments to overcome aforementioned challenges (barrier effects, transmission error, and estimation effects) in personal network size estimation were not directly utilized in this study. One is a method proposed by McCormick et al. [24] to estimate both individual social network size (i.e., degree) and the distribution of network sizes in a population by asking respondents how many people they know in specific subpopulations; this is done by using a latent non-random mixing model and a simple first name based procedure based on the model. The other is a procedure developed by Salganik et al. [25] for adjusting for transmission error by using a game-like activity called the game of contacts in order to estimate the social visibility of groups.

Despite these limitations, this study demonstrated the ability of Internet-based network scale-up methods to simply and rapidly estimate the size of hard-to-reach populations, and serves as a critically needed baseline size estimation for future behavioral sero-surveillance sample size calculations and to set HIV prevention services targets. To further improve this method for estimating hard-to-reach populations, additional refinements in survey design taking advantage of the above developments adjusted to the Japanese context to address identified limitations and verification through comparing with direct methods are needed.

### Conclusions

The results of this first study of Internet-based network scale-up methods among MSM in Japan were within the limits from those found in Japan in previous studies of MSM prevalence. Overall, size estimation through combining internet surveys with the network scale-up method appears to be an effective approach from the perspectives of rapidity, simplicity, and low cost. Using frequent and thorough size estimation of key hard-to-reach populations (such as MSM in Japan), more-effective and targeted policies should be formulated. In the field of HIV/AIDS response, for example, such policies are required if we are to realize the shared goals of zero new HIV infections, zero AIDS-related deaths and zero AIDS-related discrimination [26].

## References

[1] UNAIDS (2010). Global report: UNAIDS report on the global AIDS epidemic 2010.. http://www.unaids.org/globalreport/documents/20101123_GlobalReport_full_en.pdf.

[2] UNAIDS (2010). Global report: UNAIDS report on the global AIDS epidemic 2010.. http://www.unaids.org/globalreport/documents/20101123_GlobalReport_full_en.pdf.

[3] UNAIDS (2010). Progress reports submitted by countries.. http://www.unaids.org/en/dataanalysis/monitoringcountryprogress/2010progressreportssubmittedbycountries/japan_2010_country_progress_report_en.pdf.

[4] Mahy M (2010). Network scale-up: a promising method for national estimates of the sizes of populations at higher risk, UNAIDS quarterly update on HIV epidemiology.. http://www.unaids.org/en/media/unaids/contentassets/documents/epidemiology/2010/JC2032_20102_epialert_en.pdf.

[5] Caceres C, Konda K, Segura E, Lyerla R (2008). Epidemiology of male same sex behavior and associated sexual health indicators in low and middle-income countries: 2003–2007 estimates.. Sex Transm Infect.

[6] UNAIDS/WHO (2010). Working group on global HIV/AIDS and STI Surveillance. Guidelines on estimating the size of populations most at risk to HIV.. http://data.unaids.org/pub/Manual/2010/guidelines_popnestimationsize_en.pdf.

[7] Munakata T, Morita M (1993). Sexual intercourse between homosexuals: mode of transmission and prevention method.. Nippon Rinsho.

[8] Kihara M, Kihara M, Uchino H, Ishizuka T, Osaki Y (1999). National survey on HIV/STD knowledge, sexual behavior and awareness in Japan. Study group report on epidemiological study of HIV infection.. Health and Labour Sciences Research Grants (in Japanese).

[9] Bernard HR, Hallett T, Iovita A, Johnsen EC, Lyerla R (2010). Counting hard-to-count populations: the network scale-up method for public health.. Sex Transm Infect.

[10] Ministry of Internal Affairs and Communication (2009). State of telecommunications services usage (in Japanese).. http://www.soumu.go.jp/main_content/000064217.pdf.

[11] Hidaka Y, Ichikawa S, Koyano J, Urao M, Yasuo T (2006). Substance use and sexual behaviors of Japanese men who have sex with men: A nationwide internet survey conducted in Japan.. BMC Public Health.

[12] Fire and Disaster Management Agency (2007). The white paper on fire services (in Japanese).. http://www.fdma.go.jp/html/hakusho/h19/h19/html/j221k000.html.

[13] National Police Agency (2008). The white paper on police (in Japanese).. http://www.npa.go.jp/hakusyo/h20/honbun/pdf/20p20500.pdf.

[14] Ministry of Defense (2008). Defense of Japan (Annual white paper) (in Japanese).. http://www.clearing.mod.go.jp/hakusho_data/2008/2008/html/ks342000.html.

[15] Killworth P, Johnsen E, McCarty C, Shelley G, Bernard H (1998). A social network approach to estimating seroprevalence in the United States.. Soc Networks.

[16] Hidaka Y, Kimura H, Ichikawa S (2005). REACH online 2005..

[17] Tsuji R, Harihara M (2003). Trust relation in the ‘small world’ and social order.. Sociol Theor Method.

[18] McCarty C, Killworth P, Bernard H, Johnsen E, Shelley G (2001). Comparing two methods for estimating network size.. Hum Organ.

[19] Yasunaga H, Ide H, Imamura T, Ohe K (2004). Medical research using internet questionnaire in Japan.. Nippon Koshu Eisei Zasshi.

[20] Eysenbach G, Wyatt J (2002). Using the internet for surveys and health research.. J Med Internet Res.

[21] Kilworth P, McCarty C, Bernard H, Shelley G, Johnsen E (1998). Estimation of seroprevalence, rape and homeless in the United States using a social network approach.. Eval Rev.

[22] Kadushin C, Kilworth P, Bernard H, Beveridge A (2006). Scale-up methods as applied to estimates of heroin use.. J Drug Issues.

[23] Zheng T, Salganik MJ, Gelman A (2006). How many people do you know in prison?: Using overdispersion in count data to estimate social structure in networks.. Journal of the American Statistical Association.

[24] McCormick T, Salganik MJ, Zheng T (2010). How many people do you know?: Efficiently estimating personal network size.. Journal of the American Statistical Association.

[25] Salganik MJ, Mello M, Abdo A, Bertoni N, Fazito D (2011). The game of contacts: Estimating the social visibility of groups.. Soc Networks.

[26] UNAIDS (2010). Getting to zero: 2011–2015 strategy.. http://www.unaids.org/en/media/unaids/contentassets/documents/unaidspublication/2010/JC2034_UNAIDS_Strategy_en.pdf.

